# Identification and Pathway Analysis of microRNAs with No Previous Involvement in Breast Cancer

**DOI:** 10.1371/journal.pone.0031904

**Published:** 2012-03-16

**Authors:** Sandra Romero-Cordoba, Sergio Rodriguez-Cuevas, Rosa Rebollar-Vega, Valeria Quintanar-Jurado, Antonio Maffuz-Aziz, Gerardo Jimenez-Sanchez, Veronica Bautista-Piña, Rocio Arellano-Llamas, Alfredo Hidalgo-Miranda

**Affiliations:** 1 Laboratorio de Genómica del Cáncer, Instituto Nacional de Medicina Genómica. Mexico City, Mexico; 2 Instituto de Enfermedades de la Mama FUCAM, Mexico City, Mexico; 3 Unidad de Validación de Biomarcadores, Instituto Nacional de Medicina Genómica. Mexico City, Mexico; Centro de Investigación Príncipe Felipe, Spain

## Abstract

microRNA expression signatures can differentiate normal and breast cancer tissues and can define specific clinico-pathological phenotypes in breast tumors. In order to further evaluate the microRNA expression profile in breast cancer, we analyzed the expression of 667 microRNAs in 29 tumors and 21 adjacent normal tissues using TaqMan Low-density arrays. 130 miRNAs showed significant differential expression (adjusted P value = 0.05, Fold Change = 2) in breast tumors compared to the normal adjacent tissue. Importantly, the role of 43 of these microRNAs has not been previously reported in breast cancer, including several evolutionary conserved microRNA*, showing similar expression rates to that of their corresponding leading strand. The expression of 14 microRNAs was replicated in an independent set of 55 tumors. Bioinformatic analysis of mRNA targets of the altered miRNAs, identified oncogenes like ERBB2, YY1, several MAP kinases, and known tumor-suppressors like FOXA1 and SMAD4. Pathway analysis identified that some biological process which are important in breast carcinogenesis are affected by the altered microRNA expression, including signaling through MAP kinases and TP53 pathways, as well as biological processes like cell death and communication, focal adhesion and ERBB2-ERBB3 signaling. Our data identified the altered expression of several microRNAs whose aberrant expression might have an important impact on cancer-related cellular pathways and whose role in breast cancer has not been previously described.

## Introduction

microRNAs (miRNAs) are small non coding RNAs which regulate the expression of coding genes at a post-transcriptional level through inhibition and destabilization of messenger RNAs [1]. MicroRNAs participate in diverse biological processes like cell division, proliferation, differentiation, death, growth and development, stem cell regulation, metabolism and stress response [2], [3].

Aberrant expression of microRNAs is related with the development of different pathologies, including cancer, where they can act either as tumor suppressors or as “oncomirs” (oncogenes). Approximately 50% of the microRNA genes are located in regions commonly affected by chromosomal alterations (amplification, deletions and fragile sites) in the cancer genome [4]. As in the case of messenger RNA expression patterns, microRNA expression signatures can also be used to classify human tumors and to identify molecular signatures associated with relevant clinical characteristics [5], [6]. During the biogenesis of microRNAs, an intermediate RNA duplex represents an obligatory intermediate. After the maturation process, the mature, leading strand is preferentially incorporated into the silencing complex, while the role of the other strand (miRNA*) was regarded as a simple passenger. However bioinformatic and functional analyses have identified important regulatory roles of the miRNA*, both in normal and in pathological states, including cancer [7].

In breast cancer, analysis of microRNA expression patterns has led to the identification of signatures which can differentiate tumor from normal tissues [8], [9]. Analysis of the messenger RNA targets of microRNAs with differential expression in normal and tumor breast tissues, indicates that their aberrant expression impact the regulation of important cellular networks known to drive breast cancer [10]. This is supported by the observation that several clinically relevant breast tumor features, such as tumor size, nodal involvement, vascular invasion, hormone receptor and HER2 status, are also related to the expression of particular microRNAs [8], [9], [11]. Additionally, microRNAs might be used as markers of the metastatic potential of primary breast tumors [12].

In order to further analyze the differences in microRNA expression patterns in breast tumors, we evaluated the expression profile of 667 microRNAs in 29 breast tumors compared to 21 adjacent normal tissues. We also compared the expression patterns between four fresh frozen and their corresponding paraffin embedded tumor tissue pairs, in order to evaluate the robustness of the TaqMan low density array platform as a tool for retrospective studies.

Our analyses identified 130 differentially expressed microRNAs between the tumor and normal tissues, 43 of them whose involvement in breast cancer has not been previously described, to our best knowledge. Differential expression of 14 of these microRNAs was validated in an independent set of normal and tumor samples, suggesting that their aberrant expression might play important roles in breast cancer. Several evolutionary conserved miRNA* were included in this expression signature, showing expression rates similar to their mature strand, suggesting their potential regulatory role in breast tumors.

## Results

### MicroRNA expression profiles in breast tumors

Comparative Ct analysis (2- ΔΔCt) was used to identify a set of 130 microRNAs that were differentially expressed between the normal and tumor tissue (adjusted P value≤0.05, fold change: 2). 17 were over-expressed and 113 were down-regulated in the tumor compared to the normal tissue (Table 1 shows the microRNAs with the highest differential expression values, a complete list including all 113 is shown in Table S1). This is in accordance with other studies performed using different types of platforms, such as bead-based flow cytometry [5] or miRNA microarrays [13].

**Table 1:** MicroRNAs with the highest differential expression between normal and tumor breast tissue.

Genes	Loci	log2 fold change	Adjusted P.value
**miR-129-3p**	11p11.2	−23.86634845	0.0506
**miR-668**	14q32.31	−15.87301587	0.04
**miR-488**	1q25.2	−5.617314544	6.33E-06
**miR-204**	9q21.12	−4.752087104	4.63E-07
**miR-215**	1q41	−4.407307647	0.005803931
**miR-139-3p**	11q13.4	−3.750253537	3.49E-05
**miR-205**	1q32.2	−3.584191223	1.63E-05
**miR-654-3p**	14q32.31	−3.321708086	0.003627753
**miR-337-5p**	14q32.2	−3.27939216	0.00019613
**miR-451**	17q11.2	−3.174627414	0.001302005
**miR-504**	Xq26.3	−3.157463711	0.001762529
**miR-518b**	19q13.42	−3.153532596	0.001739482
**miR-483-5p**	11p15.5	−2.964422269	0.001296854
**miR-497**	17p13.1	−2.958883061	4.03E-05
**miR-486-3p**	8p11.21	−2.93777587	5.35E-05
**miR-145**	5q32	−2.909161828	0.001369377
**miR-543**	14q32.31	−2.874385872	2.07E-05
**miR-99a**	21q21.1	−2.842203759	0.000378898
**miR-874**	5q31.2	−2.829348053	0.000438618
**miR-100**	11q24.1	−2.781736443	7.32E-05
miR-183*	7q32.2	5.93253603	0.010473616
miR-454*	17q22	5.252342351	0.000237826
let-7g*	3p21.1	4.984783172	0.038198527
**miR-592**	7q31.33	3.914412027	0.051848409
**miR-190b**	1q21.3	3.822193954	0.011638475
**miR-449a**	5q11.2	3.546100694	0.044712337
**miR-760**	1p22.1	3.231671879	0.058593441
**miR-210**	11p15.5	2.973935913	0.016929322
miR-148b*	7p15.2	2.786665283	0.000432485
**miR-188-5p**	Xp11.23	2.55450304	0.034381478
**miR-425**	3p21.31	2.477271454	0.00683419
**miR-877**	5p15.1	2.226445287	0.010473616
miR-629*	15q23	2.188160454	0.056679644
**miR-301b**	22q11.21	2.033976085	0.036983438
**miR-636**	17q25.1	1.706601183	0.020686329
**miR-21**	17q23.1	1.271732126	0.052188036

**Figure 1:**
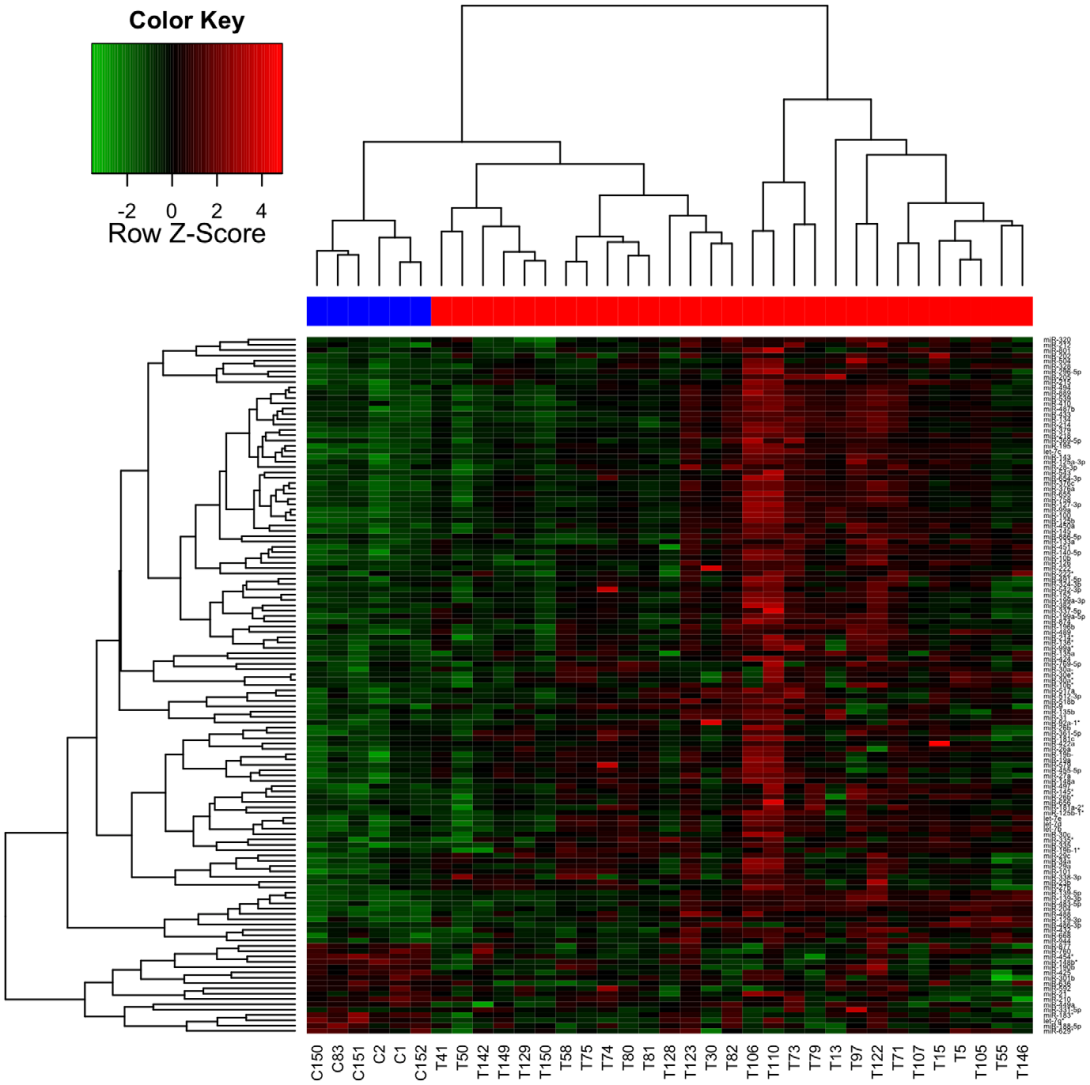
Unsupervised Hierarchical clustering analysis using the differentially expressed microRNAs separate normal and tumor breast tissues. The heatmap (Spearman correlation, Euclidean distance) represents log transformed Delta Ct values. Heat map colors correspond to microRNA expression as indicated in the color key: red (over-expressed) and green (down-regulated). Blue line: Control samples, red line: tumor samples.

Unsupervised hierarchical clustering analysis of the log-transformed delta Ct values of the differentially expressed microRNAs, showed that this set of markers is able to differentiate the tumors from the normal breast tissues (Figure 1, figure S1).

We observed an overlap between microRNAs expressed in normal and tumor samples, indicating that miRNA expression levels, rather than a differential tissue-specific pattern, is driving the separation between normal and tumor tissue.

### Identification of microRNAs with previously unknown involvement in breast cancer

**Figure 2:**
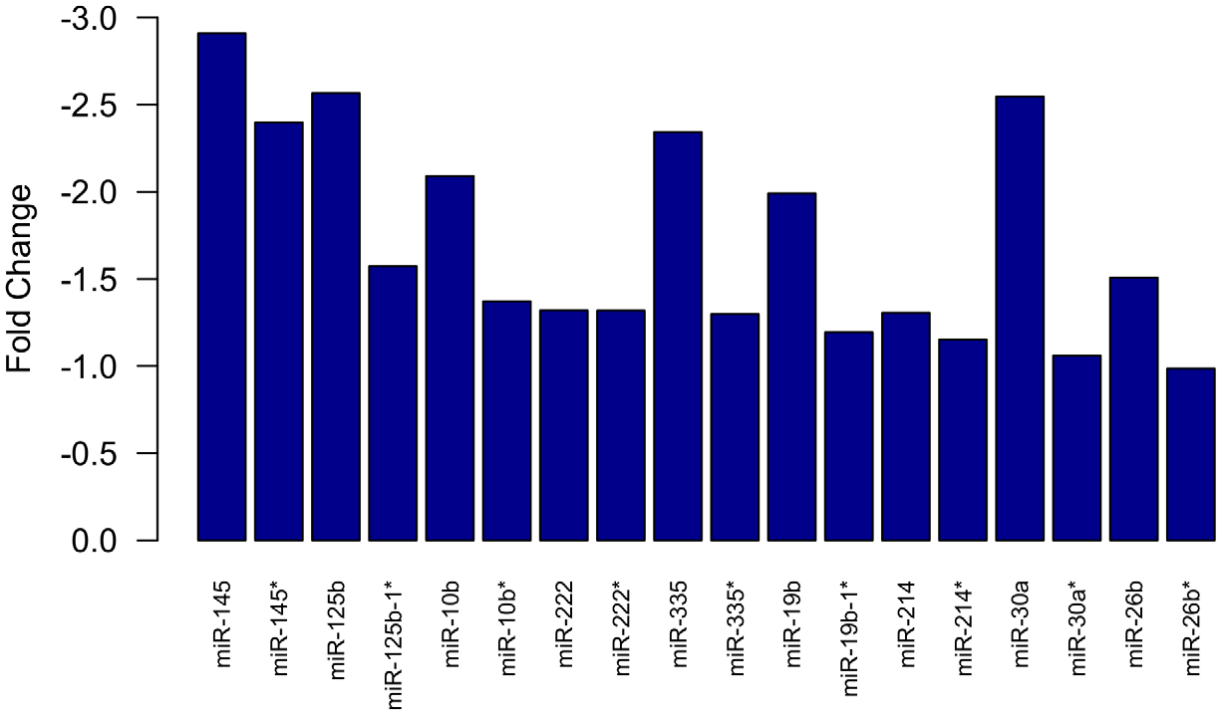
Comparison of the expression rate between miRNA-miRNA*. The bars show the normalized expression values of the microRNA pairs: driver (microRNA) and passenger (miRNA*) strands present in the breast cancer profile.

Out of the 130 differentially expressed microRNAs we detected in this study, 43 (30%) have not been previously reported in the literature as involved in breast cancer, to our best knowledge (Table 2). Some of these represent the passenger strand (miRNA*) of pre-miRNAs. In some cases, like miR-10b* and miR-145*, their corresponding leading strand have important, proven roles in breast cancer, but the role of the star strand has not been explored. Interestingly, for most of the microRNA* included in our profile, their guiding opposite strand is differentially expressed in the tumor tissue, in most cases with a very similar expression rate (Figure 2).

**Table 2:** Differentially expressed microRNAs with no previous involvement in breast cancer and their validated transcript targets.

miRNA	Validated targets	miRNA	Validated targets
**miR-379**	None reported	**miR-656**	None reported
**miR-433**	FGFR1, FGF20, HDAC6, GRB2	**miR-543**	None reported
**miR-655**	None reported	miR-10b*	DICER1, ERBB2, EGFR, RHOC
**miR-99a**	NFKB1,SLC12A2, DICER1, AKT1, MYC, IGF1	miR-145*	DGCR8, KRAS, ADAM17, BCL2, ERBB2, DICER1,
**miR-758**	None reported	miR-26b*	CCDN1, BRCA1, NOTCH1, EZH2, DICER1, MYC, EPHA2
**miR-874**	None reported	miR-30e*	ERBB2, TGFBR2, CCND1
**miR-127-3p**	BCL2, BCL6, MYC, NOTCH1,	miR-30a*	DICER1, ARL2, PRKCI, NOTCH1, SOX2
**miR-135a**	JAK2, STAT3, PI3,	miR-99a*	None reported
**miR-518b**	None reported	miR-214*	VEGFA, EZH2, MYC, PAK1, TWIST1, DICER1
**miR-382**	POMC, RUNX1, MECP2	miR-92a-1*	TGFB1, IGFR1, BRCA2, MYC
**miR-134**	CREB1, YY1, SOX2, MDM4	**miR-129-3p**	SOX4, MLH1
**miR-889**	None reported	**miR-215**	ZEB2, ALCAM, MCL-1
**miR-338-3p**	DICER1, ERCC4, HES5, MCM2, BRAF	**miR-488**	None reported
**miR-487b**	None reported	**miR-668**	None reported
**miR-455-5p**	TLR4, UCP1, MAPK3	miR-222*	ETS1, TNF, AKT1, BCL2, DICER1, EPHB2
**miR-361-5p**	TGFB1, TLR4, AKT1, IRS1	miR-454*	None reported
**miR-517a**	None reported	miR-148b*	IL6, TLR3, TLR4, APC
**miR-539**	HLCS	**miR-877**	RNASEN
**miR-512-3p**	CFLAR, CDKN1A, DNMT1	miR-183*	PIK3CA, DICER1, FOXO1, PTEN. SAG
**miR-491-5p**	FGFR1, NCOA6, FOSB, TNF	**miR-592**	DICER1
**miR-331-5p**	MDM2, SOAT1, ERBB2, JAG1	**miR-425**	DICER1, SMAD3, PDGFC
miR-136*	CD36		

The published and validated transcriptional targets (oncogenes, estrogen regulators, tumor suppressors, miRNA biogenesis machinery and epigenetic master regulators) of the miRNAs with no previous involvement in breast cancer, indicate that they might have important biological implications in breast tumor biology (Table 2).

### Evaluation of the reproducibility of the microRNA screening method

microRNA expression profiles were analyzed in a set of 29 breast tumor tissues compared to 21 normal, non paired, adjacent tissues (clinical characteristics of these samples is presented in Table 3). 23 tumors and two pools of normal samples were run in triplicate to assess the reproducibility of the TaqMan low-density array. The standard deviation between the technical triplicates was 0.1675507, while the Spearman Correlation was 97% (Max: 99%, Min: 96%). The Spearman correlation as well as the standard deviation values between our triplicates, showed a high correlation between each technical and biological replicates (Figure S2).

**Table 3:** Clinical and pathological information of the sample collection.

ID	ER	PR	Her2/neu	AGE	HISTOLOGY	STAGE	Tumor Size (cm)	TNM
**5**	−	−	+	66	IDC	IIB	3	pT2 pN1a M0
**13**	+	+	−	62	IDC	I	1.5	T1c N0 M0
**15**	−	+	+	45	IDC	IIIB	2.5	pT4b pN0 M0
**30**	+	−	−	89	IDC	IIA	1.5	T1cN0M0
**41**	−	+	−	50	IDC	I	1.5	T1N0M0
**50**	+	+	−	47	IDC	IIA	2.5	pT2 pN0 M0
**55**	+	−	+	52	IDC	I	2	pT1c pN0 M0
**58**	−	−	+	59	ISDC	IIA	3.5	pT1mic pN1mic M0
**71**	+	−	−	39	IDC	IIIA	1.9	pT1c pN2 M0
**73**	+	+	−	65	IDC	IIIA	2	pT1c pN2 M0
**74**	−	+	−	47	IMC	IIA	3.5	pT2 pN1a M0
**75**	−	−	+	50	IDC	IIB	2.5	pT2 pN1a M0
**79**	+	−	−	56	IDC	IIIA	2	pT1c pN2a M0
**80**	−	−	−	37	IDC	IIA	2.5	pT2 N0 M0
**81**	−	+	−	48	IDC	IIA	1.5	pT1c pN1a M0
**82**	+	−	−	59	IDC	IIA	2.2	pT2 pN0 M0
**97**	−	−	−	56	IDC	IIIA	4.7	pT2 pN2a M0
**105**	+	+	−	39	IDC	IIIC	2.3	pT2 N3a M0
**106**	+	+	−	42	IDC	I	1.6	pT1c pN0 M0
**107**	−	−	−	59	IDC	IIA	NA	T1>N0M0
**110**	+	−	−	39	IDC	IIB	3	T2N1Mx
**122**	−	−	−	51	IDC	IIA	1.6	pT1c pN1a M0
**123**	+	+	−	71	IDC	IIB	4.9	pT2 N1a M0
**128**	+	+	−	55	IDC	IIA	NA	T2N1M0
**129**	+	+	−	53	IDC	IIB	1.3	pT1c pN1a M0
**142**	−	+	−	47	ILC	I	1.8	T1b N0M0
**146**	−	−	−	54	IDC	IIA	NA	T2N0M0
**149**	+	−	−	61	IDC	IIB	3	T2N0M0
**150**	+	+	−	49	IDC	IIA	1.5	T2N0M0

IDC: Infiltrating ductal carcinoma, ISDC: In situ ductal carcinoma, ILC: Infiltrating lobular carcinoma, IMC: Infiltrating mucinous carcinoma, NA: Not available.

### Hormone receptor status is associated to the expression of different microRNAs

Delta Ct analysis identified differential microRNA expression signatures related with ER and PR status in the tumor samples. ER+ tumors showed differential expression of miR-342-5p, miR-29c*, miR-29b-2*, miR-30e, miR-190b, miR-769-5p, miR-30d and miR-432, with a (*P* value ≤0.05) compared to ER- tumors (Table 4, figure S3). Differential expression of miR-145*, miR-34a* and miR-193b* (adjusted *P* value ≤0.05) was able to discriminate between the PR+ from the PR- samples (Figure S4).

**Table 4:** Differentially expressed miRNAs associated with hormone receptor status.

Estrogen Receptor			
miRNA	Fold Change	p.value	Pathways of the mRNA targets
miR-342-5p	1.576048754	0.002539134	Cell cycle
miR-29c*	−2.631736726	0.007378817	
			DNA replication
miR-30e	−1.517276999	0.003742107	
			Cell surface interactions
miR-190b	3.822193954	0.001163848	
miR-30d	1.305024162	0.005495711	Apoptosis
			
miR-432	1.110681182	0.003739888	Cell cycle check points.

Progesterone Receptor			
miRNA	Fold Change	p.value	Pathways of the mRNA targets
miR-145*	3.171062506	0.043296329	Apoptosis
miR-34a*	2.12616702	0.047844819	Cell cycle Check points
			DNA repair
miR-193b*	3.125460441	0.05702715	Immune system

### Analysis of the miRNA/miRNA* sequence conservation

Most of the miRNA/miRNA* pairs included in the expression profile were conserved in the seed sequence region across the five different vertebrate genomes we analyzed (Figure S5). Some of the miRNA*, like miR-10b* and miR-30a* presented divergence in its sequence, reflected in the percentage of nucleotide substitutions. However, most of the miRNA/miRNA* showed a high degree of evolutionary conservation of the passenger miRNA* strand with a low percentage of nucleotide substitutions (miR-19b, miR-19b*, miR-125, miR-125*, miR-26b, miR-26b*, miR-145, miR-145*, miR-335, miR-335*, miR-214 and miR-214) (Figure S6). This analysis determined that the miRNA* detected in our analyses is both differentially expressed between the normal and tumor tissues, in most cases with a very similar expression pattern compared to the corresponding leading strand and is also evolutionary conserved, suggesting that they might have a biological role in breast cancer.

### Validation of differentially expressed microRNAs in an independent set of breast tumor tissues

A set of 17 microRNAs was selected for further analysis in an independent set of samples through evaluation of their expression using independent TaqMan assays. This set included 13 microRNAs with differential expression between the tumor and normal tissues and 4 with non-differential expression. Expression profiles were concordant in 14/17 (82.3%) of the selected microRNAs, and only three failed to replicate. A similar expression value was obtained for the same microRNA in both of the TLDA and the single probe assay. (Pearson Correaltion: 97.3153%) (Table 5).

**Table 5:** miRNAs selected for validation in an independent set of tumor and normal breast tissues.

	Independent Assay		TLDA		
miRNA	RQ	P-Value	RQ	Adj.P-Value	Validation
**miR-129-3p**	−22.42152466	0.0509	−23.86634845	0.0506	DR
miR-136*	−5.641025641	0.0078	−2.708003	3.85E-06	DR
**miR-99a**	−3.883495146	0.0082	−1.803093	0.013542569	DR
**miR-10b**	−5.980861244	0.0484	−2.090468209	0.000109373	DR
**miR-206**	−2.070966521	0.009	−2.355497628	0.0077	DR
**miR-27a**	−3.58347292	0.0142	−1.982850027	0.009750279	DR
**miR-145**	−2.671898854	0.04141	−2.90916182	0.001369377	DR
**miR-21**	2.2054	0.02749	1.271732126	0.052188036	OE
**miR-184**	6.672	0.2183	3.3103	0.169	NC
**miR-24**	−1.804077215	0.1159	−1.857355126	0.079	NC
**miR-492**	3.2652	0.0724	2.3677	0.1465	NC
**miR-326**	−2.419549964	0.2573	−2.578	0.678	NC
**miR-488**	−6.954102921	0.0063	−5.61731454	6.33E-06	DR*
**miR-668**	−15.87301587	0.04	−14.15451895	0.0019	DR*
miR-25*	3.4012	0.2661	20.581	0.0133	FP
**miR-431**	−8.605851979	0.1159	2.2719	0.0576	FP
miR-149*	−8.271298594	0.0033	10.269	0.0298	FP

DR: down-regulated, OR: over-expressed, DR*: down Regulated, but expressed in 78% of the samples, FP: False Positive.

### Deregulated microRNAs and their putative transcriptional targets

To define potential mRNA targets of the differentially expressed microRNAs, and their impact on cellular pathways, we performed an mRNA target prediction analysis with at least 3 different algorithms (Tables 6–7) followed by enrichment analysis of the predicted mRNA targets using Diana, mir-Path and the Reactome databases. The list of the top pathways ranked by the enrichment P-value is presented in Table 8.

**Table 6:** Differentially expressed microRNAs with no previous involvement in breast cancer and cellular pathways affected by their transcriptional targets.

miRNA	Status	Cellular pathways related with cancer
miR-129-3p	Down-regulated	Regulation of BAD phosphorylationHs_the IGF-1 Receptor and Longevity, Hs_Role of ERBB2 in signal transduction and oncology, breast cancer resistance to antimicrotubule agents and influence of RAS and RHO proteins on G1 to S Transition
miR-488	Down-regulated	CARM1 and regulation of the Estrogen Receptor
miR-139-5p	Down-regulated	Apoptosis, chromosome maintenance and transmembrane transport of small molecules.
miR-655	Down-regulated	Apoptosis, signaling by VEGF, cell cycle membrane, metabolism of protein and signaling by insulin receptor.
miR-134	Down-regulated	Transmembrane transport of small molecules and transcription
miR-136*	Down-regulated	Siganling by EGFR, signaling by Wnt, apoptosis, transcription, cell junction organization and signaling by VEGF. *
miR-874	Down-regulated	Signalling by Notch, gene expression, cell cycle, mitotic and immune system**
miR-539	Down-regulated	Apoptosis, signaling by insulin receptor, pyruvate metabolism and citric Acid (TCA) cycle, signaling of EGFR, steroid hormones, cell cycle, mitotic and signaling by Notch.
miR-491-5p	Down-regulated	DNA replication, Signaling by notch, regulatory RNA pathway and signaling by EGFR
miR-889	Down-regulated	Cell cycle, cell junction organization, membrane trafficking, metabolism of hormones and DNA repair**
miR-222*	Down-regulated	Transmembrane transport of small molecules, apoptosis, cell cycle, signaling by EGFR and cell cycle checkpoint ***
miR-877	Over-expressed	Signaling by EGFR and cell junctions Organization
miR-425	Over-expressed	EXT2 (possible tumor suppressor), MET receptor, PAK4. Cell cycle signaling, cell signaling checkpoints and transport of small molecules and biological oxidation.
miR-454*	Over-expressed	Transmembrane transport of small molecules, membrane trafficking, apoptosis, cell cycle, and DNA replication*
miR-592	Over-expressed	ERBB4, CD200, ST7. DNA replication, apoptosis, mRNA processing, transcription and metabolism of lipids
let-7g*	Over-expressed	Cell junction organization, apoptosis, Immune system, signaling by TGF beta and integration of energy metabolism**
miR-183*	Over-expressed	Transmembrane transport of small molecules, cell junction organization, signaling by VEGFR, and integration of energy metabolism**

* mRNA targets predicted only with Miranda,

** mRNA targets predicted with Miranda and Targetscan,

*** mRNA targets predicted with Miranda and Pictar.

**Table 7:** Previously reported miRNAs with differential expression in breast cancer, some of their mRNA targets and the cellular pathways where they participate.

miRNA	Status	Targets and cellular pathways related with cancer	Bibliography
miR-125a/b	Down-regulated	Oncogene: ERBB2 and ERBB3. Signal transduction: MAP3K10 and MAP3K11. c-raf-1	[32]
miR-10b	Down-regulated	Growth factor: FLT and BDNF. Transducing factor :SHC1. Oncogene: Rho. Homeobox gene: HOXD10	[13]
let-7	Down-regulated	Oncogene: RAS. Architectural factor: HMGA2	[13]
miR-205	Down-regulated	Growth factor: TGF-β. HER3 phenotype. Oncogene: ErbB3 and Zeb1	[61]
miR-145	Down-regulated	Kinase: RAF1. Oncogene: YES	[13], [62]
miR-31	Down-regulated	Oncogene: Rho, Metastasis-promoting genes FZAD3, ITGA5, M-RIP. Regulate invasion and metastasis. Patients with higher miR-31 expression, had prolonged survival.	[63]
miR-335	Down-regulated	Oncogene: SOX4 and TNC. TNC (Tenascin C): responsible for the acquisition of metastatic properties	[64]
miR-126	Down-regulated	Growth factor: VEGF. Insulin receptor tyrosine kinase: IRS-1	[64]
miR-101	Down-regulated	EZH2, oncogenic and metastatic activity	[65]
miR-206	Down-regulated	CARM1 and Regulation of the Estrogen Receptor, T Cell Receptor Signaling Pathway, Regulation of BAD phosphorylation.	[66]
miR-222	Down-regulated	ERα negatively modulates miR-222. Confers proliferative advantage and migratory activity to cells and promote the transition from ER-positive to ER-negative tumors	[32]
miR-21	Over-expressed	BCL-2, PTEN, MASPIN, involved in apoptosis	[38]
miR-210	Over-expressed	Overexpression is induced by hypoxia in a HIF-1α– and VHL-dependent manner. miR-210 influences the hypoxia response by targeting a transcriptional repressor of the MYC-MAX pathway (6)	[67]

**Table 8:** Statistically significant biological pathways potentially affected by the differentially expressed microRNAs in breast cancer.

Pathway	# of Genes (Union)	p-value	Description (KEGG Pathway Database)
MAPK signaling pathway	130	0.0002488	Involved in cellular functions: cell proliferation, differentiation and migration.
Wnt signaling pathway	85	0.0000056	Required for developmental processes, cell proliferation and cell division.
Focal adhesion	100	0.000000193	Cell motility, cell proliferation, cell differentiation, regulation of gene expression and cell survival
Adherens junction	47	0.0001819	Important for maintaining tissue architecture and cell polarity
TGF-beta signaling pathway	54	0.00001657	Regulates proliferation, apoptosis, differentiation and migration
Insulin signaling pathway	73	0.00001462	Allows Tyrosine phosphorylation of insulin receptor substrates
Regulation of actin cytoskeleton	100	0.0001411	Cell-matrix adhesions: cell motility, cell proliferation, cell differentiation, regulation of gene expression and cell survival
ErbB signaling pathway	49	0.0001177	Regulates proliferation, differentiation, cell motility, and survival.
Ubiquitin mediated proteolysis	66	0.0000034	Signal for protein degradation
Gap junction	48	0.000826	Intercellular channels to communicate the cytosolic compartments with neighbor cells
Basal cell carcinoma	29	0.0000621	Common cancer related with epithelia
Calcium signaling pathway	71	0.0000547	Electrochemical gradient across the plasma membrane
VEGF signaling pathway	34	0.000047	Mediator of VEGF-driven responses, is a crucial signaling pathologic angiogenesis
Androgen and estrogen metabolism	8	0.0000361	Sexual hormones
Glycerophospholipid metabolism	31	0.0000344	Lipid metabolism
Hedgehog signaling pathway	26	0.0000332	Regulates stem cell proliferation
Cell Communication	33	0.000000329	Intercommunication between cells
Jak-STAT signaling pathway	59	0.0000213	Signaling mechanism of cytokines and growth factors
Tight junction	52	0.00186	Mediate cell adhesion
TP53 signaling pathway	28	0.00000157	Responses to stress signals, DNA damage, oxidative stress and activated oncogenes

### Comparison of microRNA profiles between Paraffin embedded and fresh-frozen tissues

**Figure 3:**
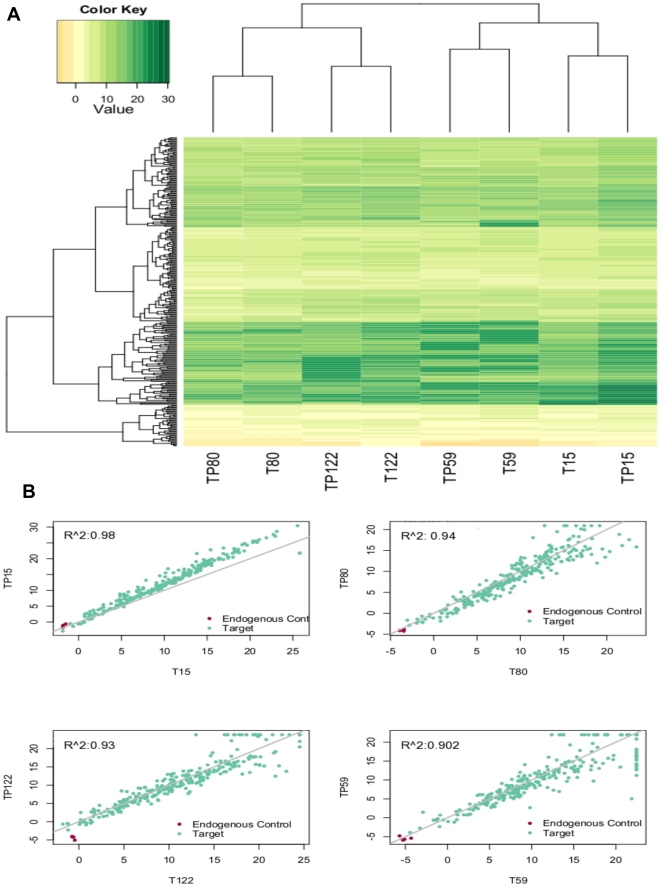
Correlation between FFPE and fresh samples. A) Unsupervised hierarchical cluster of the delta Ct values of all the analyzed microRNAs represented as a Heat Map. B) Scatter plot of the Ct values in pair-wise comparisons from fresh (T) and FFPE samples (TP).

Formalin fixed paraffin embedded (FFPE) tissue represents a major source of potentially useful biological material for retrospective analysis. To determine the performance and robustness of the microRNA TLDA system in the analysis of microRNAs obtained from FFPE tissues, we compared the expression patterns of pairs of fresh-frozen and FFPE tissues from the same patient. Correlation between these results was analyzed using Spearman correlation and unsupervised hierarchical clustering. We observed cluster aggregation, as well as a high correlation value between the fresh and the FFPE tissues obtained from the same patient (average 93.75%, minimum of 90%; maximum of 98%), indicating that results obtained from RNA isolated from FFPE tissues retain the same expression signature as the fresh frozen tissue (Figure 3).

## Discussion

### Differentially expressed microRNAs with no previous involvement and potential relevance in breast cancer

We have analyzed the microRNA expression patterns in a set of breast cancer tumors and compared them to normal tissue. Our analyses identified a set of differentially expressed microRNAs whose role in breast cancer has not been previously described, to our best knowledge. Several evolutionary conserved miRNA* were included in this expression signature, showing expression rates similar to their mature strand. Differential expression of a set of these microRNAS was validated in an independent set of tumor and normal breast tissues, suggesting their potential regulatory role in breast tumors.

Finally, we evaluated the performance of the TaqMan low-density array through the comparison of microRNA expression patterns obtained from fresh-frozen and FFPE tissue pairs, obtaining similar results in both cases.

Between the microRNAs without previous involvement in breast cancer, we identified the down-regulation of miR-129-3p. In mouse lung epithelial cells, lentiviral mediated expression of miR-129-3p results in G1 phase arrest and cell death through down-regulation of CDK6, ERK1 and ERK2, indicating its activity in cell proliferation. Epigenetic repression of miR-129 also leads to over-expression of the SOX1 oncogene in gastric and endometrial cancer [14]. In breast cancer, SOX1 is activated due to the loss of miR-335, which was also found down-regulated in our study (Table 1) suggesting that miR-129 might also play a role in the SOX1 mediated acquisition of metastatic capacity [12], [15].

Decreased expression of miR-215, down-regulated in our expression profile, has been associated with cell proliferation rate and has been proposed as a tumor suppressor candidate in colon cancer. miR-215 reduces cell proliferation and cell cycle G2-arrest through regulation of dihydrofolate reductase thymidylate synthase (DTL), and increased expression of TP53 and p21 [16]. DTL has been implicated in cell proliferation, cell cycle arrest and cell invasion in diverse tumor types, including hepatocellular carcinoma and breast cancer [17].

We found down-regulation of some of the miR-99 family members, including miR-99a, this has also been described in advanced prostate cancer cell lines and tissues. The direct targets of the miR-99 family are the chromatin remodeling factors SMARCA5, SMARCD1 and the growth regulatory kinase mTOR, which is also an important pathway activated in breast cancer [18]. miR-99a, has also been found downregulated in serous ovarian carcinoma [19].

Senescence represents a potent tumor suppressive mechanism, and involvement of microRNAs in this process has been already described. miR-668, down-regulated in head and neck squamous cell carcinoma cell lines and in our expression profile, has been identified as a senescence-inducing microRNAs, playing an important role not only in cancer pathogenesis, but also as an interesting target for the development of new therapeutic targets [20].

Over-expression of mir-425 has been described in several human cancer cells [21] and its pri-miRNA sequence is evolutionarily conserved in different mammals (human, mouse, dog and opossum) supporting the idea that miR-425 plays a regulatory role in eukaryotic cells [22]. TargetScan, Mirtarget2, Miranda, miRTar, PITA and RNA hybrid algorithms, predicted DICER1 and SMAD2 as potential targets of miR-425. SMAD is involved in the regulation of DROSHA, another key player in small RNA processing [23], indicating that aberrant expression of miR-425 might have important effects in the biogenesis of small RNAs.

miR-592 was over-expressed in our tumor dataset. This microRNA has been found differentially expressed between DNA mismatch repair deficient and proficient colon tumors. The interactions between miR-592 and genes associated with the mismatch repair system suggest an oncogenic role of this miRNA, possibly acting through inhibition of tumor suppressor genes [24]. Upregulation of miR-592 has also been reported as part of the microRNA signature of kidney cancer [25].

miR-877 is a DROSHA independent intronic microRNA, up-regulated in our breast tumor samples. It is part of the coding region of the ATP-binding cassette subfamily F member 1 (ABCF1), which is over expressed in breast cancer. ABCF1 is a transporter of molecules through membranes, and has been associated to drug resistance and the development of some types of cancer [26].

Together with this set of novel microRNAs, we also confirmed the differential expression of microRNAs whose role and biological targets in breast cancer have been well described (Table 5). This is the case of the down-regulation of miR-125b [13], [27], let-7 [5], [13], [28] miR-205 [29]–[31], miR-145 [13], [31], miR10b [13], miR-222 [32], miR34a [33], miR-31[34], miR-206 [12], [13], [35]; and over-expression of miR-210 [13], [36], [37] and miR-21 [13], [28], [38] (check reference [39] for a review).

As part of our expression profile we found an important presence of miRNAs*. Recent bioinformatic and experimental data show a high degree of conservation over vertebrate evolution, particularly in the seed regions of expressed miRNAs* [7]. The miRNA/miRNA* ratios also change in different developmental stages [40] and have been involved in the regulation of different biological networks in normal physiological conditions [41]. In cancer, miRNA* expression has been detected in childhood acute lymphoblastic leukemia [42], myelodysplastic syndrome [43], cell lines of tumors of the female reproductive tract [44] and melanoma [45] indicating their potential role in cellular transformation. The set of miRNA* we found differentially expressed between the normal and tumor breast tissues show a high degree of evolutionary conservation, according to our analysis in five animal genomes, as well as a similar expression rate than their corresponding leading strand. These results suggest that these miRNA* might be playing regulatory roles in breast cancer.

Breast cancer etiology includes genomic alterations that drive cancer cell development, like loss of heterozygosity, amplifications, deletions and fragile sites, which can promote oncogene activity or repress the expression of tumor suppressors [46]. More than half of the human miRNAs (60%) are located in regions commonly affected in the cancer genome, a situation that might affect their expression [4]. Our analysis identified down-regulation of 22 microRNAs located in the 14q32 region, which is deleted in approx. 10% of breast tumors [20], and has been reported as a chromosomal region where several breast cancer-related microRNAs are located [47] (Figure S7), suggesting that loss of this chromosomal region and the down-regulation of the miRNAs codified in this locus, might be correlated in a fraction of breast tumors.

Hormone receptor status is an important tumor characteristic to classify breast cancer and to determine clinical treatment. However, there is limited information about the genetic mechanisms regulating the expression of hormone receptors. We identified a set of miRNAs which can differentiate hormone receptor positive and negative tumors. Analysis of the mRNA targets of these miRNAs identified biological pathways relevant to breast cancer, like apoptosis, DNA repair, cell cycle check-points, etc. (Table 3). The miRNAs in the ER signature can directly regulate transcripts like ESR1 (miR-342-5p, miR-190b, miR-432), ESRRG (miR-30d, miR-30e), ESRRA (miR-432); ERN2 (miR-342-5p), which induces translational repression in response to ER stress, coactivators of the estrogen receptor like PELP1 (miR-342-5p) and SRC (miR-342-5p); mediators of cell cycle trough estrogen activation like E2F (miR-30d, miR-30e and miR-432) and transcription factors like DP1 (miR-30e). While miRNAs of the PR profile can regulate the activity of the progesterone receptors PRDM4 (miR-34a*), PRDM16 (miR-145*) and co-activators of the progesterone pathway like SRC (miR-34*, mir-145*) and MAPK (miR-145*, miR-193*, miR-34*). miR-190 and miR-345 have already been reported as discriminators of ER status [47], suggesting its importance in the establishment of this phenotype.

Deregulation of microRNA expression might potentially affect the regulation of multiple cancer-related genes; for this reason it's important to define the biological networks affected by differentially expressed microRNAs and their transcriptional targets. Pathway analysis of our expression profile determined different transcripts and protein-protein interactions, which can be activated or repressed by these microRNAs. Examples of these pathways are ERBB signaling, which plays a determinant role in breast cancer [48] through its contribution to tumor development, cellular transformation, involvement in the development of central nervous system metastases and targeted therapy [49]. Another important pathway affected by the differentially expressed miRNAs is the mitogen-activated protein kinase (MAPK), which is activated in breast cancer and is involved in the initiation and pathogenesis of breast tumors [50]. Analysis of the targets affected by the miRNAs with no previous relation with breast cancer included in our signature, also identified several cancer-related pathways, including KRAS, EGFR, MAPK, VEGF, ERBB, PTEN, FOS, AKT1, etc. (Table 6, table 8, figure S8).

Finally, in order to evaluate the potential application of the TLDA platform in the retrospective evaluation of miRNA expression patterns in breast cancer, we carried out a comparison between results obtained from fresh frozen and FFPE tissue. Results of the comparison showed a high correlation between the two tissues, indicating that the platform can be used in retrospective studies using FFPE tissue [51].

## Materials and Methods

### Breast tissue samples and RNA extraction

After obtaining the written patient's informed consent, tumor and normal adjacent breast samples were collected during surgery at the Institute of Breast Diseases (FUCAM) in Mexico City. The protocol was reviewed and approved by the Ethics and Research committees of the National Institute of Genomic Medicine and the Institute of Breast Diseases in Mexico City (CE2009/11). Tissues were macroscopically analyzed by a trained pathologist and stored at −80°C until further processing. Frozen sample sections were evaluated histologically to assure that only samples with more than 80% of tumor cells were included in our analyses. Frozen tissues were disrupted with a Tissue Ruptor (Qiagen Inc., Valencia, CA) and total RNA was obtained using the Trizol protocol (Invitrogen, Carlsbad, CA). For the FFPE tissues, total RNA was isolated with the Recoverall kit (Ambion, Austin, USA) according to the manufacture's protocol. Briefly, 10-8 µm sections were incubated in xylene for 3 minutes at 50°C for de-paraffinization, followed by two brief washes in 100% ethanol. Once ethanol was evaporated, RNA extraction was done as described in the kit's protocol. Total RNA concentration was evaluated by spectrofotometry (NanoDrop Technologies, Wilmington, Delaware). Total RNA integrity from the frozen samples was analyzed using the Agilent 2100 Bioanalyzer with the Nano-Eukariotic chip.

### MicroRNA expression analysis

The Megaplex TLDA, v2.0 (TaqMan® Low Density Array, Applied Biosystems (ABI), Foster City, CA) platform was used to measure miRNA expression. There are two plates in this system: plate A, containing well-characterized and widely expressed microRNAs, while plate B presents less characterized microRNAs. The combined plates evaluate the expression of 667 unique human specific microRNAs (present in V14 of the Sanger miRBase) in parallel. Briefly, the procedure begins with the retro-transcription of 70 ng of total RNA with stem-loop primers to obtain a cDNA template. A pre-amplification step was included in order to increase the concentration of the original material and to detect microRNAs that are expressed at low levels. The pre-amplified product was loaded into the TaqMan® Low Density Arrays and amplification signal detection was carried out using the 7900 FAST real time thermal cycler (ABI). A total of 29 tumor and 21 normal samples (two pools: one containing five normal samples, other containing 12 normal samples, plus 4 independent normal samples) were analyzed. 23 tumors and the two normal pools were processed by triplicate, representing 82% of the total samples. Raw miRNA expression data is available at the Gene Expression Omnibus (GEO), with accession number GSE35412.

### Statistical analysis

To determine the expression level of each miRNA, the comparative Ct (2^−ΔΔCt^) method was used. RNU44 and RNU48 showed the most stable expression between samples and were selected for normalization across all experiments [52]. All analyses were done using R (HTqPCR, gplots-bioconductor). The Ct raw data (fractional cycles numbers at which the fluorescence cross the threshold) was determined using an automatic baseline and a threshold of 0.2. Samples with a Ct value of <36 cycles were excluded from the analyses and the normal tissue samples were used as calibrators. A geometric mean was used to obtain the media between replicates and the outliers among replicates were excluded. A 2-fold change value obtained by the comparative Ct method (2^−ΔCT^) was used to determine the differentially expressed microRNAs. An adjusted t-test was used to evaluate the significance differences in the Ct values between controls and tumors as well as between hormone receptor positive and negatives tumors. Only microRNAs with an adjusted *P* value of 0.05, a fold change of 2 and consistent expression in at least 80% of the samples were considered as differentially expressed. Unsupervised clustering analysis, using Spearman correlation and average linkage, was used to identify different sub-groups defined by miRNA expression profiles. The rank-invariant normalized data was evaluated through Spearman correlation analysis between technical and biological replicates.

### Analysis of potential mRNAs targeted by differentially expressed microRNAs

Possible mRNA targets of the differentially expressed microRNAs were identified using the mirDIP (2011) [53] and miRwalk databases (2011) [54], through an integrative evaluation with different algorithms: TargetScan v5.1 (http://www.targetscan.org), PicTar (http://pictar.mdc-berlin.de), miRanda (www.microrna.org/microrna/getGeneForm.do), and Pita (genie.weizmann.ac.il/pubs/mir07/mir07_data.html). We only considered as potential mRNA targets those who were predicted by three of these algorithms. Gene ontology and cellular pathway analysis altered by the aberrant expression of the microRNAs was done using the Reactome [55] and DIANA lab software [56], which obtained the information from TargetScan 5 (2009) [57], PicTar 4-way (2007) [58], and visualized in Wikipathways [59].

### Biological replication of differentially expressed microRNAs

Independent RT-PCR analysis using specific TaqMan microRNA assays was performed to replicate the expression of 17 microRNAs (miR-10b, miR-668, miR-431, miR-136*, miR-129-3p, miR-488, miR-99a, miR25*, miR-27a, miR149*, miR-206, miR492, miR-21) in an independent set of 20 normal and 55 fresh-frozen breast tumor samples. All assays were run in duplicate. These microRNAs were chosen based on differential expression between tumors and normal tissues and statistical confidence. Of this set of miRNAs, there is no previous information regarding the expression of miR- 136*, miR-99a, miR-488 and miR-668, in breast tumors. MiR-184, miR-24, miR-492 and miR-326, whose expression did not changed significantly between tumors and normal tissues in the TLDA assay, were selected as experimental controls, while RNU-44 and RNU-48 were used as endogenous controls for normalization.

### Conservation of mature miRNA/miRNA* sequence analysis

We made a comprehensive computational survey of miRNA conservation sequence across 5 animal genomes: *Gasterosteus aculeatus* (fish), *Xenopus tropicalis* (frog), *Anolis carolinensis* (reptile), *Gallus gallus* (bird), *Monodelphis domestica* (marsupial), *Mus musculus* (rodent) and Homo sapiens. We used the miROrtho database [60], to make multiple ortholog alignments and evaluate the conservation of the RNA secondary structure. For this analysis, we blasted the hairpin structure sequence of each miRNA obtained from miRBase.

### Correlation between miRNA profiles in FFPE tissues and fresh frozen samples

Four fresh-frozen samples and their corresponding FFPE tissues obtained from the same patient's tumor were analyzed with the TLDA platform (plate A) to evaluate the effect of formalin fixation and paraffin embedding process on the microRNA expression patterns. Correlation between the fresh-frozen and the FFPE results was evaluated using Spearman Correlation. For all statistical analysis, the log transformed delta Ct values were used. Non-supervised clustering analysis was done using Euclidian distance and average linkage including all samples. The results were visualized in a heat map.

In conlusion, our analysis identified a set of microRNAs with no previously known involvement in breast cancer, whose altered expression target relevant cellular pathways. The identification of a set of evolutionary conserved microRNA* showing differential expression between the normal and tumor tissues interesting research opportunities to study the role of the passenger microRNA strand in cancer.

## Supporting Information

Figure S1 **Principal Component analysis based in the miRNA differential expression profile.** The two most informative components were plotted. Clustering of the normal tissues and tumor tissues is observed.
(TIF)

Figure S2 **Signal correlation Plot between the biological and technical samples analyzed.** A) Scarlet plots of the correlation between expression values between control samples evaluated by Spearman correlation (correlation: 100-93%) B) Scarlet plots of the correlation between expression values between breast tumor tissues (correlation: 100-84%).
(TIF)

Figure S3 **Unsupervised hierarchical clustering using the differentially expressed miRNAs between Estrogen Receptor (ER) positive and ER negative samples.** The heatmap (Spearman correlation, Euclidean distance, complete linkage) represents Delta Ct values. Heat map colors correspond to miRNA expression as indicated in the color key: red over-expressed and green down-regulated. Salmon line: ER negative and Dark blue line: ER positive.
(TIF)

Figure S4 **Unsupervised hierarchical clustering using the differentially expressed miRNAs in Progesterone Receptor (PR) positive and PR negative samples.** The heatmap (Spearman correlation, Euclidean distance, complete linkage) represents Delta Ct values. Heat map colors correspond to miRNA expression as indicated in the color key: red over-expressed and green down-regulated. Salmon line: PR negative and Dark blue line: PR positive.
(TIF)

Figure S5 **Analysis of evolutionary conservation by multiple sequence aligments.** The upper panel shows sequence alignments with the consensus hairpin sequence and the conservation profile displayed in the grey histogram. The mature miRNA sequence is underlined. The miRNA sequence is located at the left side of the aligned sequences while the miRNA* is at the right. The inferior panel shows the consensus secondary structure of the orthologous sequence. The color-coding of the nucleotide changes is shown in the box.
(TIF)

Figure S6 **Consensus secondary structure of the orthologous sequence.** Percentage of the miRNAs nucleotide subtitutions in each miRNA/miRNA* of the seed regions (2–8 nucleotide). The blue bars represents the miRNA strand, the red bars represents the miRNA* strand.
(TIF)

Figure S7 **Chromosome 14 and policistronic miRNAs.** A) Number of miRNAs included in the expression profile and their chromosomal location. Asterisks indicate chromosomes with the higher numbers of differentially expressed miRNAs. B) microRNAs with differential expression in chromosome 14. Green lines indicate the miRNAs that are included in our differential profile.
(TIF)

Figure S8 **Gene Ontology analysis of the pathways affected by the differentially expressed novel microRNAs.** Enrichment analysis made with the mRNA targets of the not previously reported miRNAs. Yellow circles indicate the pathways associated with breast cancer.
(TIF)

Table S1 **Complete list of the 130 differentially expressed miRNAs between normal and breast tumors.**
(DOC)
