# Correction: Identification and Pathway Analysis of microRNAs with No Previous Involvement in Breast Cancer

**DOI:** 10.1371/journal.pone.0137738

**Published:** 2015-09-02

**Authors:** Sandra Romero-Cordoba, Sergio Rodriguez-Cuevas, Rosa Rebollar-Vega, Valeria Quintanar-Jurado, Antonio Maffuz-Aziz, Gerardo Jimenez-Sanchez, Veronica Bautista-Piña, Rocio Arellano-Llamas, Alfredo Hidalgo-Miranda

The following information is missing from the Funding section: This paper is part of the PhD thesis project of S. Romero-Cordoba, a student of the Biomedical Sciences PhD Program of the Autonomous National University of Mexico.

The complete, correct Funding statement is: This work was funded by the National Institute of Genomic Medicine (INMEGEN/CI/019/2009). S. Romero-Cordoba, RRV, and RAL received a PhD scholarship from the Mexican National Council of Science and Technology. This paper is part of the PhD thesis project of S. Romero-Cordoba, a student of the Biomedical Sciences PhD Program of the Autonomous National University of Mexico. The funders had no role in study design, data collection and analysis, decision to publish, or preparation of the manuscript.
