# Supplementary figures and images for: Identification and Pathway Analysis of microRNAs with No Previous Involvement in Breast Cancer
Source: PLoS One. 2012 Mar 16;7(3):e31904. doi: 10.1371/journal.pone.0031904 (PMC3306365; doi:10.1371/journal.pone.0031904)

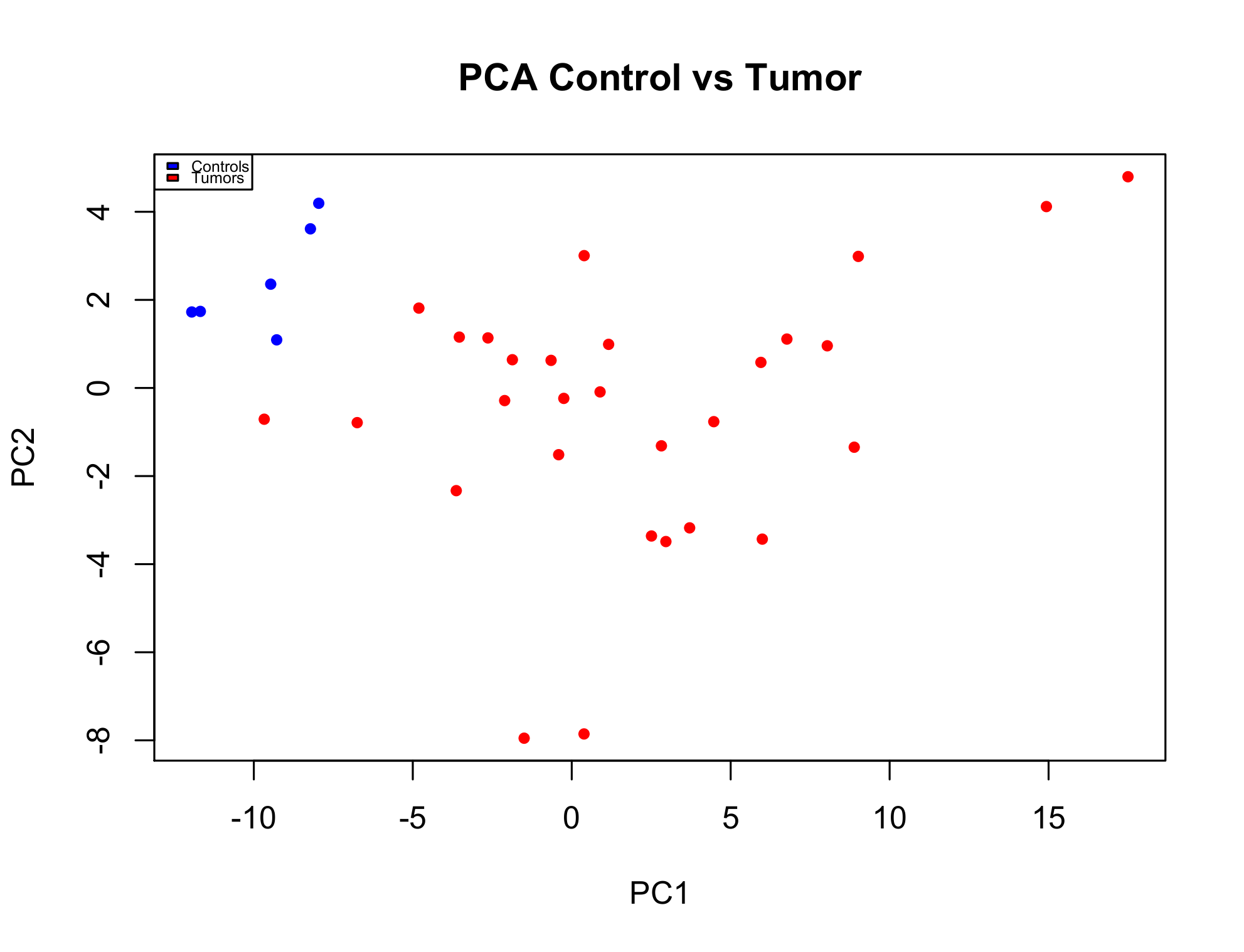

Supplement: Figure S1 — Principal Component analysis based in the miRNA differential expression profile. The two most informative components were plotted. Clustering of the normal tissues and tumor tissues is observed. (TIF) [file pone.0031904.s001.tif]

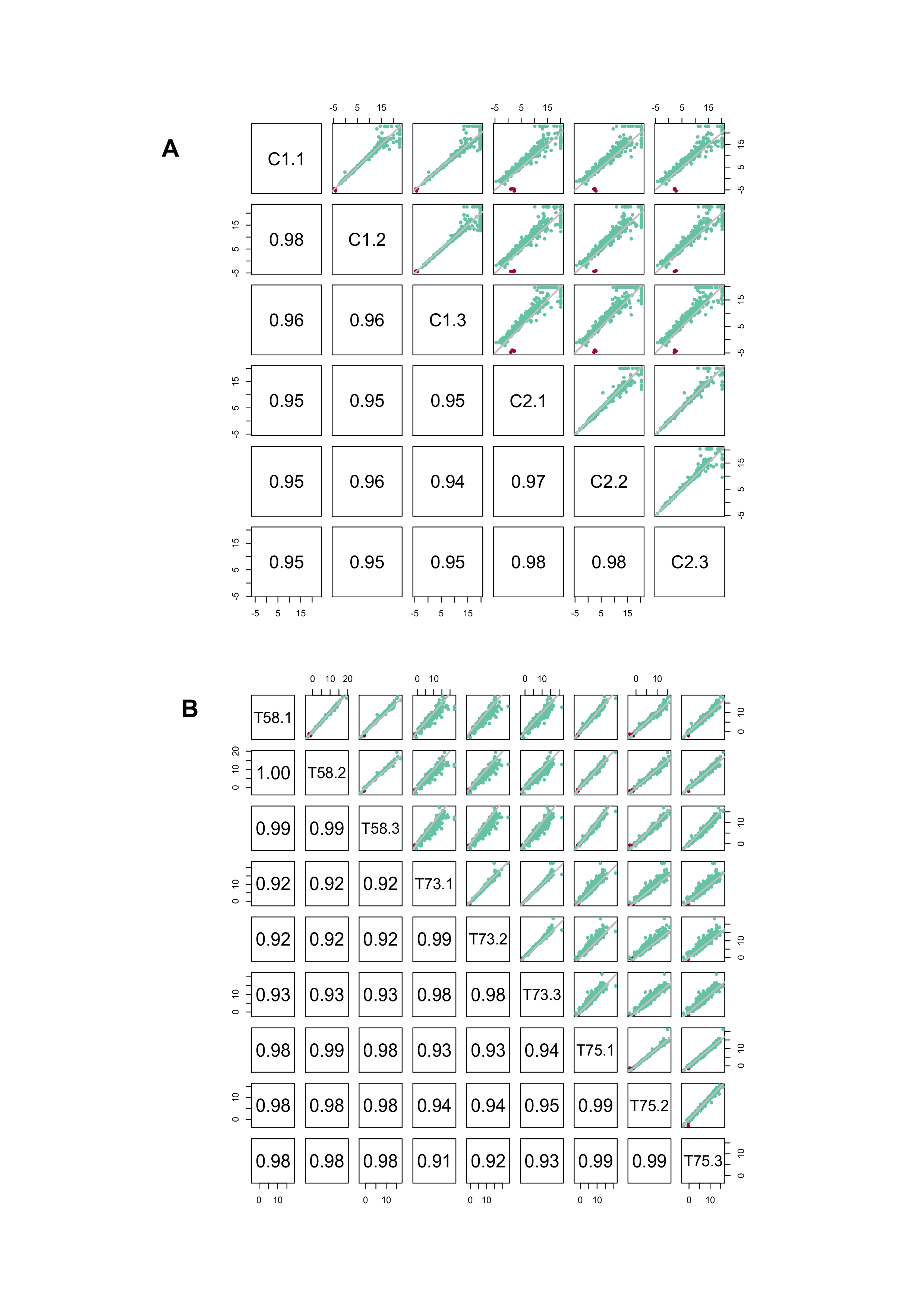

Supplement: Figure S2 — Signal correlation Plot between the biological and technical samples analyzed. A) Scarlet plots of the correlation between expression values between control samples evaluated by Spearman correlation (correlation: 100-93%) B) Scarlet plots of the correlation between expression values between breast tumor tissues (correlation: 100-84%). (TIF) [file pone.0031904.s002.tif]

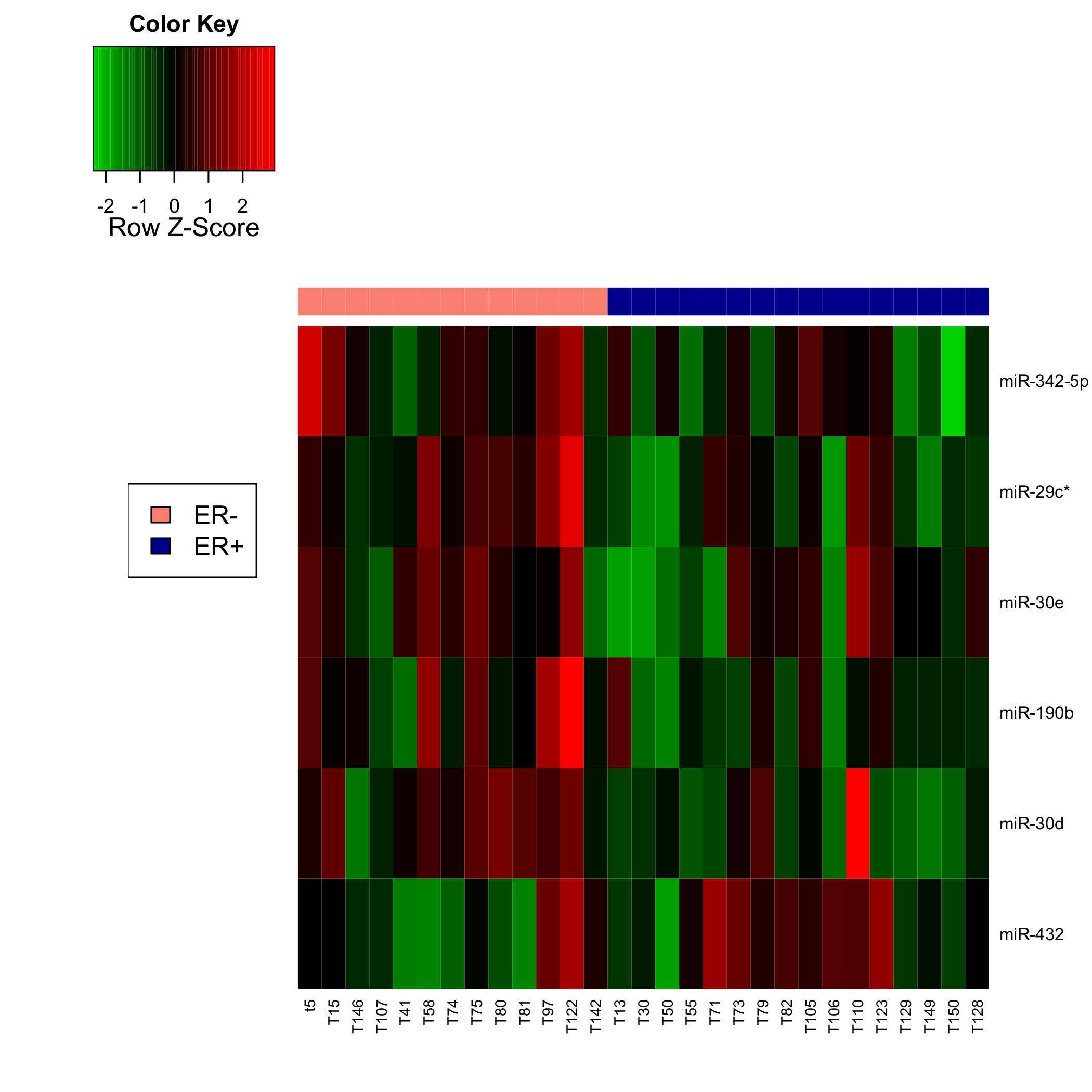

Supplement: Figure S3 — Unsupervised hierarchical clustering using the differentially expressed miRNAs between Estrogen Receptor (ER) positive and ER negative samples. The heatmap (Spearman correlation, Euclidean distance, complete linkage) represents Delta Ct values. Heat map colors correspond to miRNA expression as indicated in the color key: red over-expressed and green down-regulated. Salmon line: ER negative and Dark blue line: ER positive. (TIF) [file pone.0031904.s003.tif]

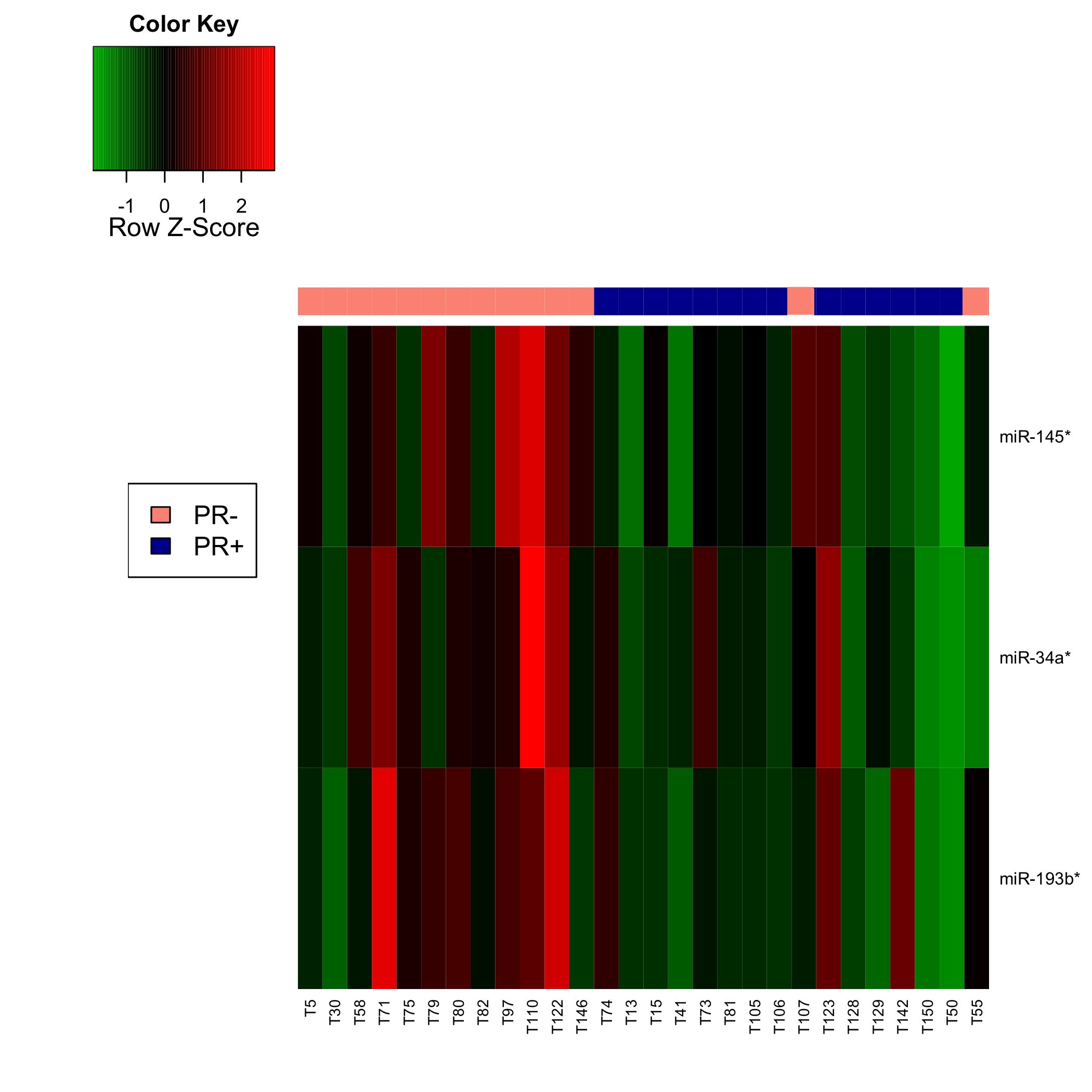

Supplement: Figure S4 — Unsupervised hierarchical clustering using the differentially expressed miRNAs in Progesterone Receptor (PR) positive and PR negative samples. The heatmap (Spearman correlation, Euclidean distance, complete linkage) represents Delta Ct values. Heat map colors correspond to miRNA expression as indicated in the color key: red over-expressed and green down-regulated. Salmon line: PR negative and Dark blue line: PR positive. (TIF) [file pone.0031904.s004.tif]

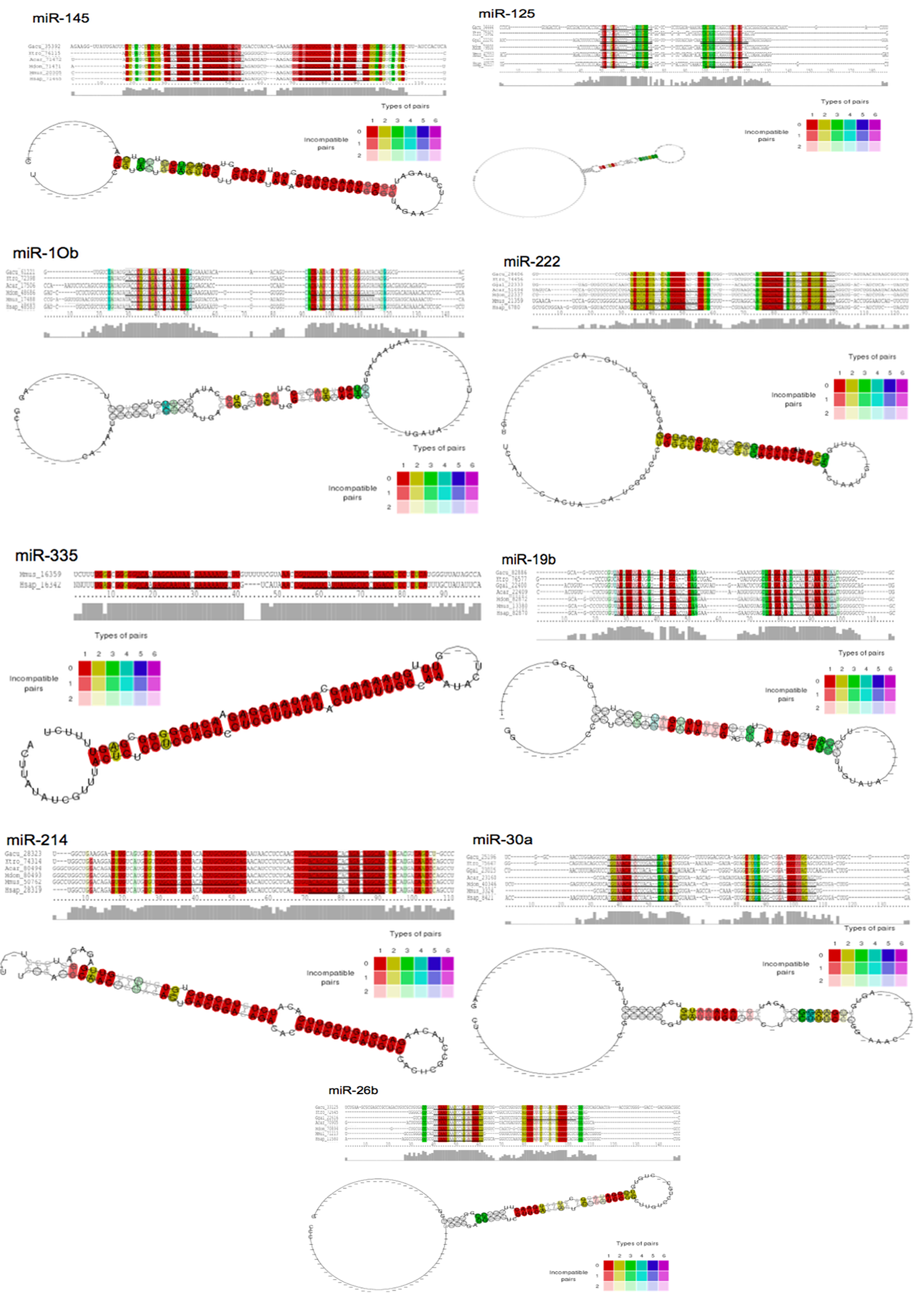

Supplement: Figure S5 — Analysis of evolutionary conservation by multiple sequence aligments. The upper panel shows sequence alignments with the consensus hairpin sequence and the conservation profile displayed in the grey histogram. The mature miRNA sequence is underlined. The miRNA sequence is located at the left side of the aligned sequences while the miRNA* is at the right. The inferior panel shows the consensus secondary structure of the orthologous sequence. The color-coding of the nucleotide changes is shown in the box. (TIF) [file pone.0031904.s005.tif]

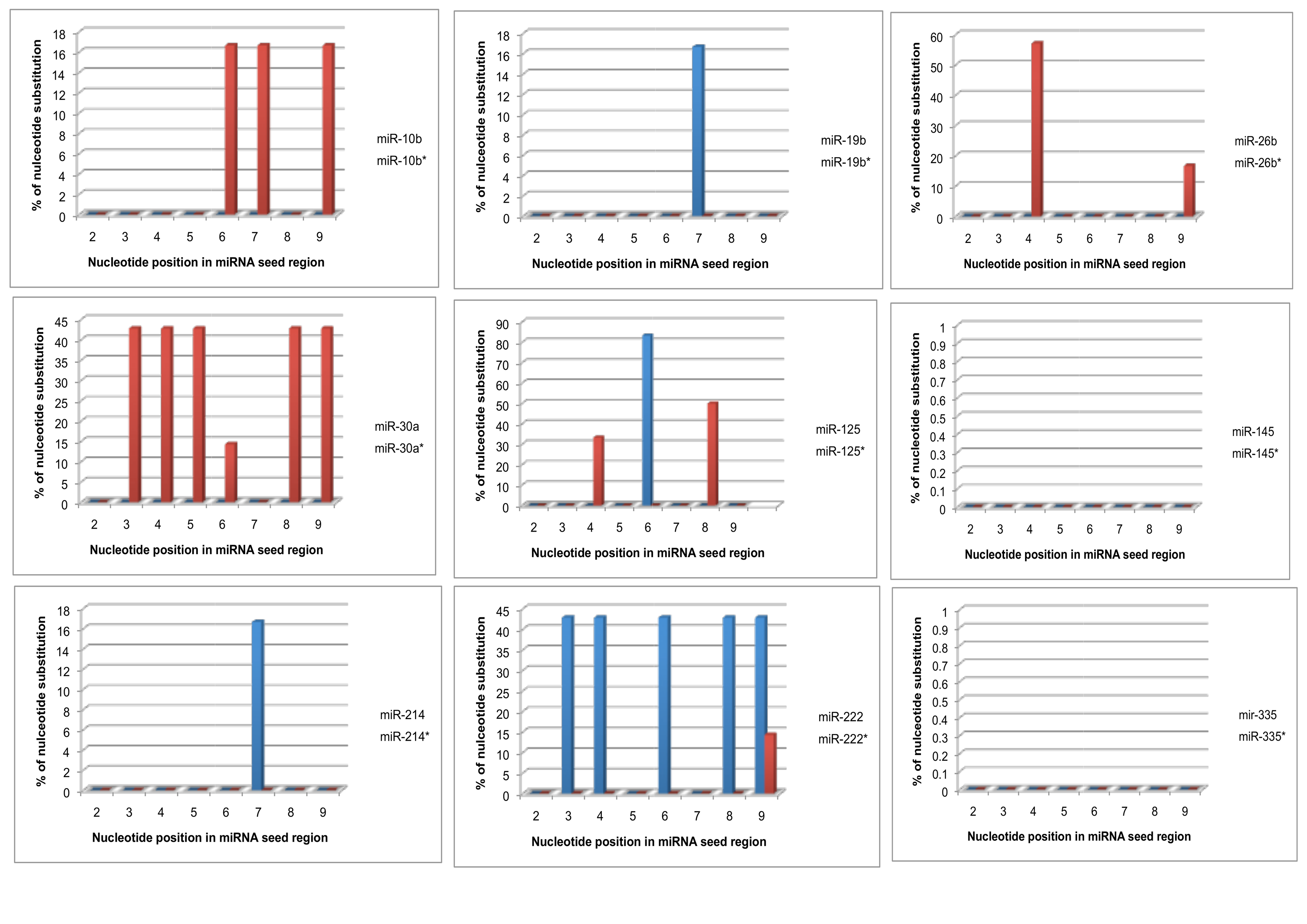

Supplement: Figure S6 — Consensus secondary structure of the orthologous sequence. Percentage of the miRNAs nucleotide subtitutions in each miRNA/miRNA* of the seed regions (2–8 nucleotide). The blue bars represents the miRNA strand, the red bars represents the miRNA* strand. (TIF) [file pone.0031904.s006.tif]

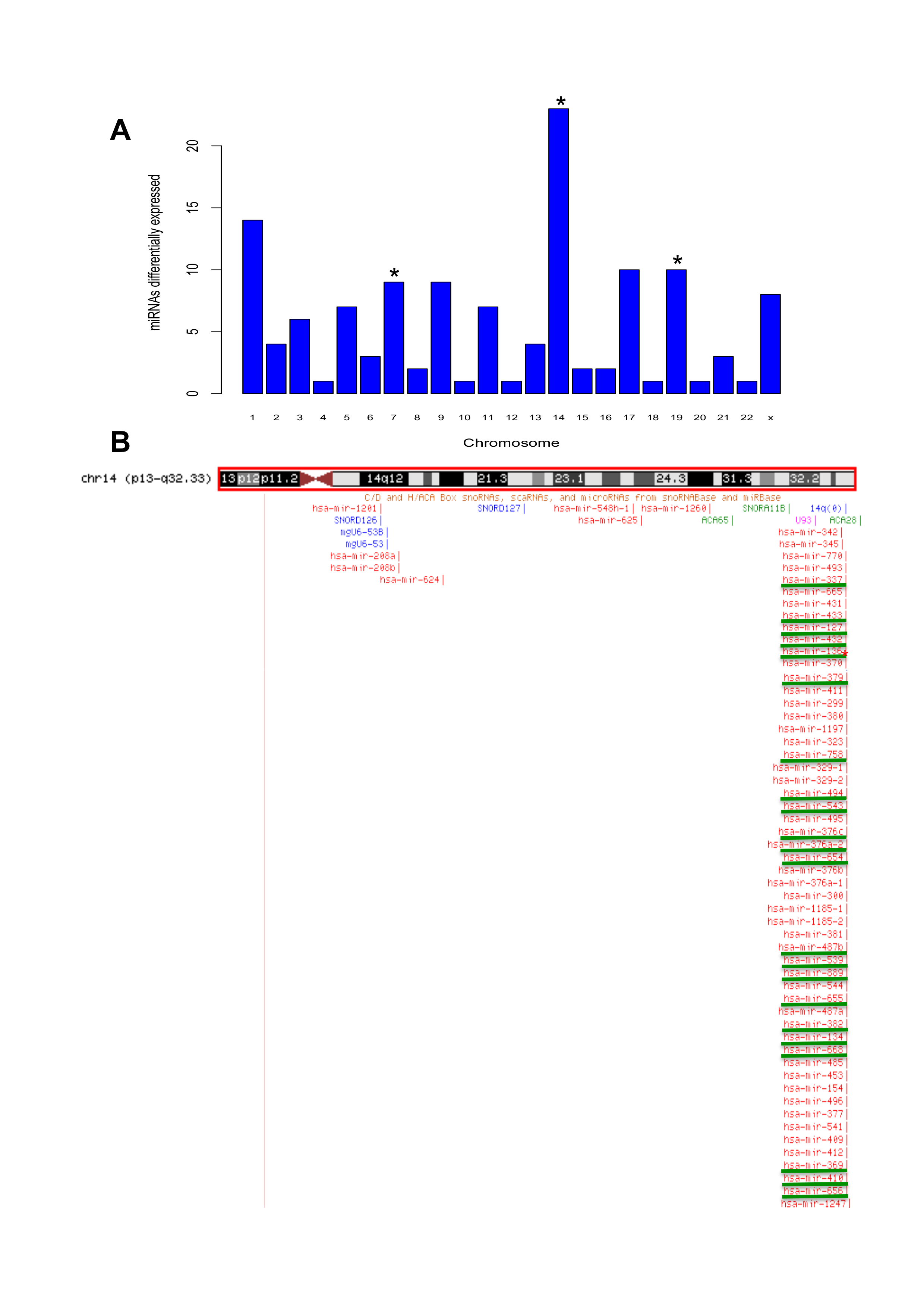

Supplement: Figure S7 — Chromosome 14 and policistronic miRNAs. A) Number of miRNAs included in the expression profile and their chromosomal location. Asterisks indicate chromosomes with the higher numbers of differentially expressed miRNAs. B) microRNAs with differential expression in chromosome 14. Green lines indicate the miRNAs that are included in our differential profile. (TIF) [file pone.0031904.s007.tif]

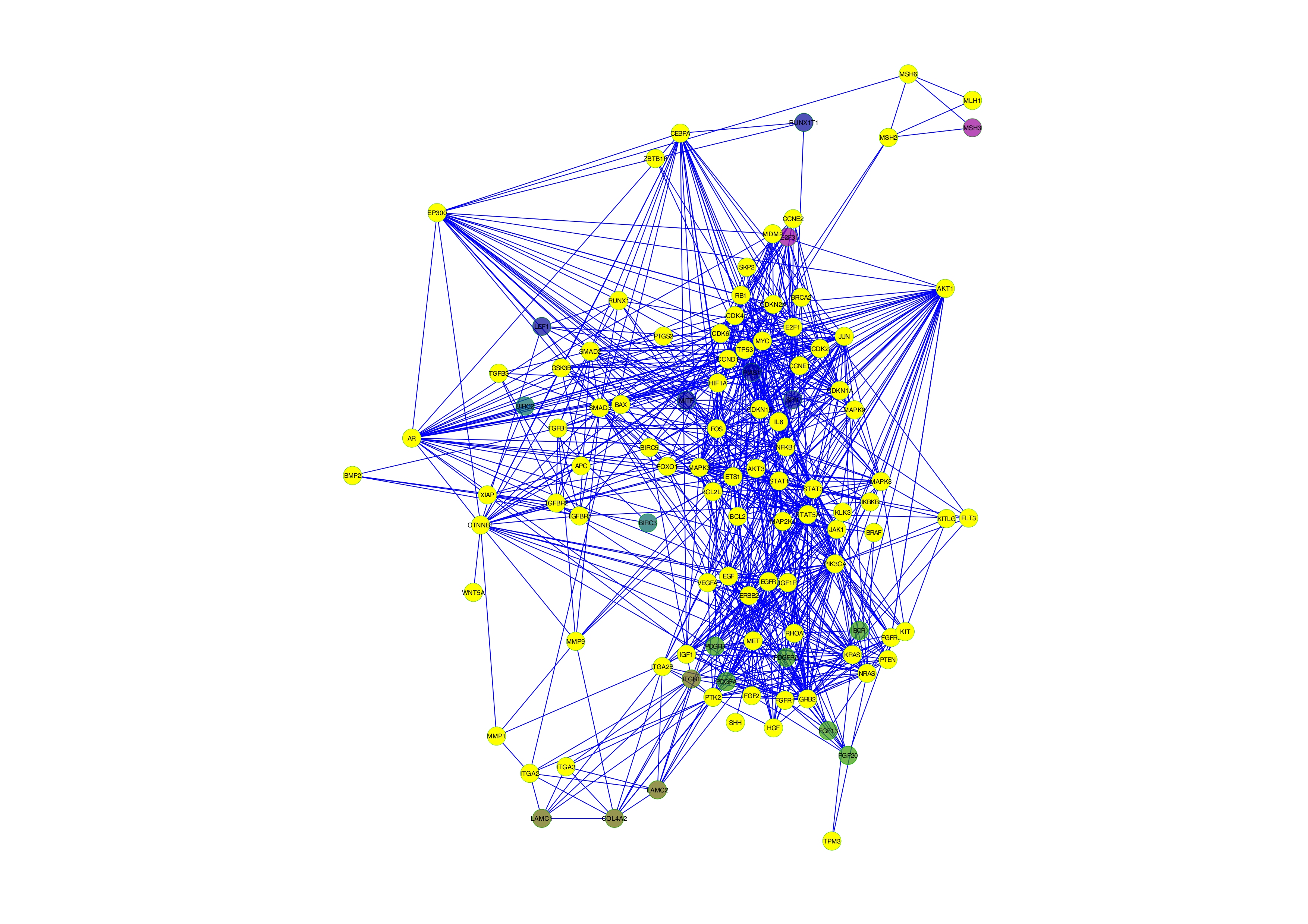

Supplement: Figure S8 — Gene Ontology analysis of the pathways affected by the differentially expressed novel microRNAs. Enrichment analysis made with the mRNA targets of the not previously reported miRNAs. Yellow circles indicate the pathways associated with breast cancer. (TIF) [file pone.0031904.s008.tif]
