# Supplementary material for: Identification and Pathway Analysis of microRNAs with No Previous Involvement in Breast Cancer
Source: PLoS One. 2012 Mar 16;7(3):e31904. doi: 10.1371/journal.pone.0031904 (PMC3306365; doi:10.1371/journal.pone.0031904)
Supplement: Table S1 — Complete list of the 130 differentially expressed miRNAs between normal and breast tumors. (DOC) [file pone.0031904.s009.doc]

**Table S1.**

**A)**

**C)**

**B)**

**D)**

S1:

| Genes | Loci | log2 FC | Adj pvalue | Genes | Loci | log2 FC | Adj pvalue |
| --- | --- | --- | --- | --- | --- | --- | --- |
| miR-129-3p | 11p11.2 | -23.86634845 | 0.0506 | miR-140-5p | 16q22.1 | -1.766405401 | 0.008726031 |
| miR-668 | 14q32.31 | -15.87301587 | 0.04 | miR-450a | Xq26.3 | -1.764502143 | 0.000209719 |
| miR-488 | 1q25.2 | -5.617314544 | 6.33E-06 | miR-101 | 1p31.3 | -1.660989263 | 0.022437697 |
| miR-204 | 9q21.12 | -4.752087104 | 4.63E-07 | miR-512-3p | 19q13.42 | -1.642719704 | 0.029617848 |
| miR-215 | 1q41 | -4.407307647 | 0.005803931 | miR-539 | 14q32.31 | -1.602845425 | 0.020686329 |
| miR-139-3p | 11q13.4 | -3.750253537 | 3.49E-05 | miR-26a | 3p22.2 | -1.591979977 | 0.013450645 |
| miR-205 | 1q32.2 | -3.584191223 | 1.63E-05 | miR-125b-1* | 11q24.1 | -1.572730769 | 0.012484532 |
| miR-654-3p | 14q32.31 | -3.321708086 | 0.003627753 | miR-134 | 14q32.31 | -1.560497104 | 0.00311814 |
| miR-337-5p | 14q32.2 | -3.27939216 | 0.00019613 | miR-29a | 7q32.2 | -1.560322472 | 0.00595956 |
| miR-451 | 17q11.2 | -3.174627414 | 0.001302005 | miR-338-3p | 17q25.3 | -1.543182318 | 0.008947926 |
| miR-504 | Xq26.3 | -3.157463711 | 0.001762529 | miR-26b | 2q35 | -1.508031841 | 0.018730089 |
| miR-518b | 19q13.42 | -3.153532596 | 0.001739482 | let-7e | 19q13.41 | -1.503901717 | 0.02262419 |
| miR-483-5p | 11p15.5 | -2.964422269 | 0.001296854 | miR-30c | 1p34.2 | -1.488302455 | 0.023405026 |
| miR-497 | 17p13.1 | -2.958883061 | 4.03E-05 | miR-455-5p | 9q32 | -1.487605476 | 0.012572121 |
| miR-486-3p | 8p11.21 | -2.93777587 | 5.35E-05 | miR-424 | Xq26.3 | -1.483591908 | 0.00480342 |
| miR-145 | 5q32 | -2.909161828 | 0.001369377 | miR-199a-3p | 19p13.2 | -1.452569574 | 0.008008459 |
| miR-543 | 14q32.31 | -2.874385872 | 2.07E-05 | miR-133a | 18q11.2 | -1.449087703 | 0.005672349 |
| miR-99a | 21q21.1 | -2.842203759 | 0.000378898 | miR-27b | 9q22.32 | -1.444295312 | 0.015061742 |
| miR-874 | 5q31.2 | -2.829348053 | 0.000438618 | miR-542-3p | Xq26.3 | -1.416177317 | 0.001710777 |
| miR-100 | 11q24.1 | -2.781736443 | 7.32E-05 | miR-410 | 14q32.31 | -1.415136298 | 0.007271555 |
| miR-136* | 14q32.2 | -2.708003272 | 3.85E-06 | miR-199a-5p | 19p13.2 | -1.384976049 | 0.041221761 |
| miR-517a | 19q13.42 | -2.684858667 | 0.014238603 | miR-19a | 13q31.3 | -1.381937471 | 0.047974357 |
| miR-376c | 14q32.31 | -2.657127878 | 7.32E-05 | miR-10b* | 2q31.1 | -1.37188953 | 0.000889828 |
| let-7c | 21q21.1 | -2.653987198 | 0.000392414 | let-7d | 9q22.32 | -1.335185715 | 0.021760788 |
| miR-23b | 9q22.32 | -2.630092711 | 0.008947926 | miR-222 | Xp11.3 | -1.319412304 | 0.006544536 |
| miR-487b | 14q32.31 | -2.604941326 | 0.010198014 | miR-222* | Xp11.3 | -1.318724792 | 0.043156701 |
| miR-139-5p | 11q13.4 | -2.592992732 | 3.20E-06 | miR-212 | 17p13.3 | -1.31782966 | 0.048107779 |
| miR-125b | 11q24.1 | -2.566057741 | 0.000209719 | miR-125a-3p | 19q13.41 | -1.307450346 | 0.00595956 |
| miR-655 | 14q32.31 | -2.549387534 | 0.000273577 | miR-214 | 1q24.3 | -1.305778937 | 0.00097759 |
| miR-30a | 6q13 | -2.546672 | 0.0005676 | miR-335* | 7q32.2 | -1.297908739 | 0.026811095 |
| miR-135a | 3p21.2 | -2.479971202 | 0.001651246 | miR-361-5p | Xq21.2 | -1.285815085 | 0.013566493 |
| miR-143 | 5q32 | -2.445023758 | 0.002272144 | miR-126 | 9q34.3 | -1.205027348 | 0.002272144 |
| miR-489 | 7q21.3 | -2.444277798 | 0.00683419 | miR-152 | 17q21.32 | -1.193797666 | 0.003486314 |
| miR-31 | 9p21.3 | -2.419316756 | 0.001568158 | miR-19b-1* | 13q31.3 | -1.193785498 | 0.005021588 |
| miR-376a | 14q32.31 | -2.414299275 | 6.96E-05 | miR-34a | 1p36.22 | -1.168477284 | 0.078693418 |
| miR-296-5p | 20q13.32 | -2.413845695 | 0.000392414 | miR-214* | 1q24.3 | -1.15184857 | 0.020150913 |
| miR-145* | 5q32 | -2.397813946 | 0.001373767 | miR-30e* | 1p34.2 | -1.135140127 | 0.008652466 |
| miR-656 | 14q32.31 | -2.388631468 | 2.04E-05 | miR-432 | 14q32.2 | -1.110681182 | 0.003739888 |
| miR-379 | 14q32.31 | -2.385066842 | 3.20E-06 | miR-196b | 7p15.2 | -1.064018209 | 0.025165927 |
| miR-335 | 7q32.2 | -2.343467414 | 0.002272144 | miR-30a* | 6q13 | -1.059445701 | 0.011672885 |
| miR-494 | 14q32.31 | -2.340382151 | 0.001184821 | miR-181a-2* | 9q33.3 | -1.020851443 | 0.013542569 |
| miR-195 | 17p13.21 | -2.32212135 | 7.32E-05 | miR-26b* | 2q35 | -0.986087802 | 0.007439277 |
| miR-382 | 14q32.31 | -2.307545205 | 0.002333236 | miR-491-5p | 9p21.3 | -0.982760137 | 0.047974357 |
| miR-369-5p | 14q32.31 | -2.282602177 | 0.00480342 | miR-181c | 19p13.13 | -0.945171126 | 0.02262419 |
| miR-422a | 15q22.31 | -2.187563235 | 0.047974357 | miR-769-5p | 19q13.32 | -0.911321061 | 0.011638475 |
| miR-328 | 16q22.1 | -2.184911222 | 0.00311814 | miR-324-3p | 17p13.1 | -0.874310529 | 0.006690081 |
| miR-758 | 14q32.31 | -2.166860982 | 0.000379611 | miR-28-3p | 3q28 | -0.813114337 | 0.019294881 |
| miR-889 | 14q32.31 | -2.123191601 | 0.00683419 | miR-320 | 8p21.3 | -0.666077059 | 0.011073534 |
| miR-127-3p | 14q32.2 | -2.122857883 | 0.000698973 | miR-331-5p | 12q22 | -0.273153265 | 0.584302118 |
| miR-202 | 10q26.3 | -2.119206538 | 0.003390027 | miR-183* | 7q32.2 | 5.93253603 | 0.010473616 |
| miR-218 | 4p15.31 | -2.112265206 | 8.06E-06 | miR-454* | 17q22 | 5.252342351 | 0.000237826 |
| miR-10b | 2q31.1 | -2.090468209 | 0.000109373 | let-7g* | 3p21.1 | 4.984783172 | 0.038198527 |
| miR-206 | 6p12.2 | -2.070966521 | 0.0077 | miR-592 | 7q31.33 | 3.914412027 | 0.051848409 |
| miR-944 | 3q28 | -2.008726988 | 0.008652466 | miR-190b | 1q21.3 | 3.822193954 | 0.011638475 |
| miR-19b | 13q31.3 | -1.991366725 | 0.012408024 | miR-449a | 5q11.2 | 3.546100694 | 0.044712337 |
| miR-27a | 19p13.13 | -1.982850027 | 0.009750279 | miR-760 | 1p22.1 | 3.231671879 | 0.058593441 |
| miR-433 | 14q32.2 | -1.980493648 | 0.00019613 | miR-210 | 11p15.5 | 2.973935913 | 0.016929322 |
| miR-9 | 1q22 | -1.943418186 | 0.021483134 | miR-148b* | 7p15.2 | 2.786665283 | 0.000432485 |
| miR-148a | 7p13.2 | -1.894067741 | 0.011073534 | miR-188-5p | Xp11.23 | 2.55450304 | 0.034381478 |
| miR-135b | 1q32.1 | -1.862762418 | 0.029452758 | miR-425 | 3p21.31 | 2.477271454 | 0.00683419 |
| let-7b | 22q13.31 | -1.819138974 | 0.003382074 | miR-877 | 5p15.1 | 2.226445287 | 0.010473616 |
| miR-29c | 1q32.2 | -1.803492886 | 0.02482674 | miR-629* | 15q23 | 2.188160454 | 0.056679644 |
| miR-99a* | 21q21.1 | -1.803093908 | 0.013542569 | miR-301b | 22q11.21 | 2.033976085 | 0.036983438 |
| miR-92a-1* | 13q31.3 | -1.796640464 | 0.030382641 | miR-636 | 17q25.1 | 1.706601183 | 0.020686329 |
| miR-579 | 5p13.3 | -1.78856623 | 0.00480342 | miR-21 | 17q23.1 | 1.271732126 | 0.052188036 |
